# Epstein-Barr Acute Viral Hepatitis With Hyperferritinemia Presents as Obstructive Cholangitis

**DOI:** 10.7759/cureus.54614

**Published:** 2024-02-21

**Authors:** Dhaval Trivedi, Julia Szinte, Sara Hasan, Samir K Shah, Sabrina Saleem

**Affiliations:** 1 Internal Medicine, New York Presbyterian - Brooklyn Methodist Hospital, Brooklyn, USA

**Keywords:** ebv-associated hepatitis, epstein-barr virus, hepatitis, ebv, myocarditis

## Abstract

Epstein-Barr Virus (EBV), also known as human herpesvirus 4, is a rare cause of hepatitis and myocarditis. Severe cases of EBV hepatitis have been documented in immunocompromised cases; however, it is even more uncommonly seen in the immunocompetent population. Our case highlights EBV hepatitis presenting as acute abdominal pain in a young male with no known medical conditions.

## Introduction

Epstein-Barr Virus (EBV) is a double-stranded DNA virus that infects B lymphocyte cells. It has infected over 95% of adults throughout the world and is the causative agent in infectious mononucleosis; treatment is generally supportive care. Identifying EBV in infected patients is most effective through serological testing in addition to atypical-appearing lymphocytes on peripheral blood smear. Heterophile antibody tests identify IgM antibodies against EBV (sensitivity 63-84%, specificity 84-100%) [1]. EBV is usually self-limiting and has a good prognosis, as most patients improve with time; however, EBV may be associated with various complications.

The diagnosis of EBV hepatitis is suggested with appropriate clinical symptoms and laboratory findings of positive EBV IgM antibody, monospot, and/or heterophile antibody testing. Other overlapping clinical presentations and potential causes of viral hepatitis should be ruled out, including but not limited to cytomegalovirus, varicella-zoster virus, herpes simplex virus, hepatitis A/B/C, and human immunodeficiency virus. Notably, EBV hepatitis often presents as asymptomatic elevations in transaminase levels, up to 2-3 times the upper limit of normal. In some instances, patients may exhibit more significant elevations (5-10 times the upper limit of normal), with a minority (<5%) presenting with jaundice. It is treated with supportive care with most cases of hepatitis resolving spontaneously. Steroids and antiretroviral therapies have been used in severe cases of EBV hepatitis, but randomized studies have not yet been performed to examine cause-effect relationships [2]. Considering the rarity and non-specific presentation of EBV hepatitis, it is important to keep this diagnosis as a differential in a similar patient presentation to properly manage these patients and prevent unnecessary workup and therapy.

## Case presentation

A 37-year-old male with no past medical history presented to the emergency department with one day of sharp intermittent right lower quadrant abdominal pain associated with five days of intermittent fevers, relieved with ibuprofen. One day before presentation, he experienced worsening nausea with non-bloody non-biliary emesis followed by mild epigastric pain. On the day of presentation, the patient began to have severe intermittent right lower quadrant abdominal pain, described as cramping. During this time, the patient endorsed decreased appetite, dysuria, and dark-colored urine. He denied jaundice, scleral icterus, or stool color changes. He was immediately seen by the general surgical service in the emergency room for concern of possible cholecystitis.

Upon physical examination, he was febrile to 39.1C with sinus tachycardia at 111 beats/min and normotensive. The abdomen was soft, non-distended, and non-tender to palpation.

Laboratory studies showed elevated aspartate transaminase (AST), alanine transaminase (ALT), and total bilirubin with slight elevations in lipase (Table 1).

**Table 1:** Initial Patient Laboratory Values

Laboratory Value	Patient	Reference Range
White Blood Cell (x 10^3^/uL)	8.42	3.5-11.0
Hemoglobin (g/dL)	15.6	11.5-14.5
Platelets (x 10^3^/uL)	188	150-400
Aspartate Transaminase (U/L)	247	<40
Alanine Transaminase (U/L)	282	<40
Alkaline Phosphatase (U/L)	197	<200
Total Bilirubin (mg/dL)	3.2	0.1-1.2
Direct Bilirubin (mg/dL)	2.7	0.0-0.3

**Figure 1:**
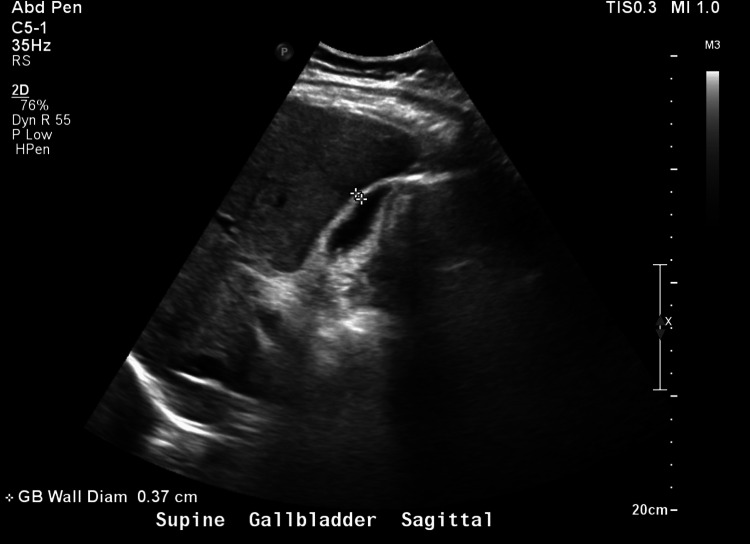
Ultrasound Abdomen Right Upper Quadrant - Gallbladder

**Figure 2:**
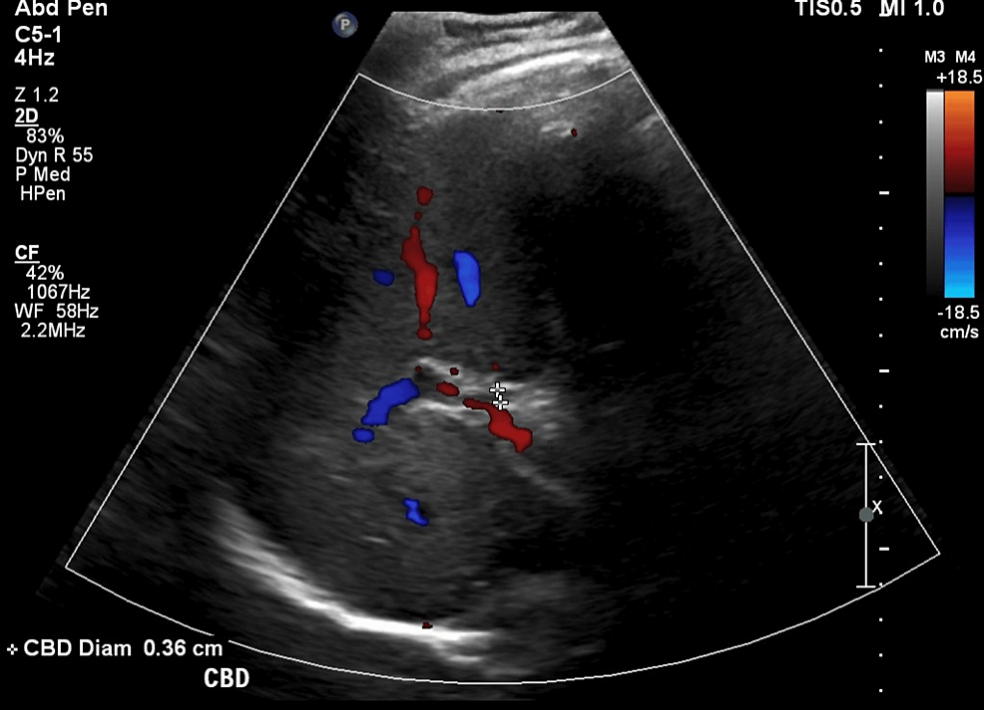
Ultrasound Abdomen Right Upper Quadrant - Common Bile Duct

**Figure 3:**
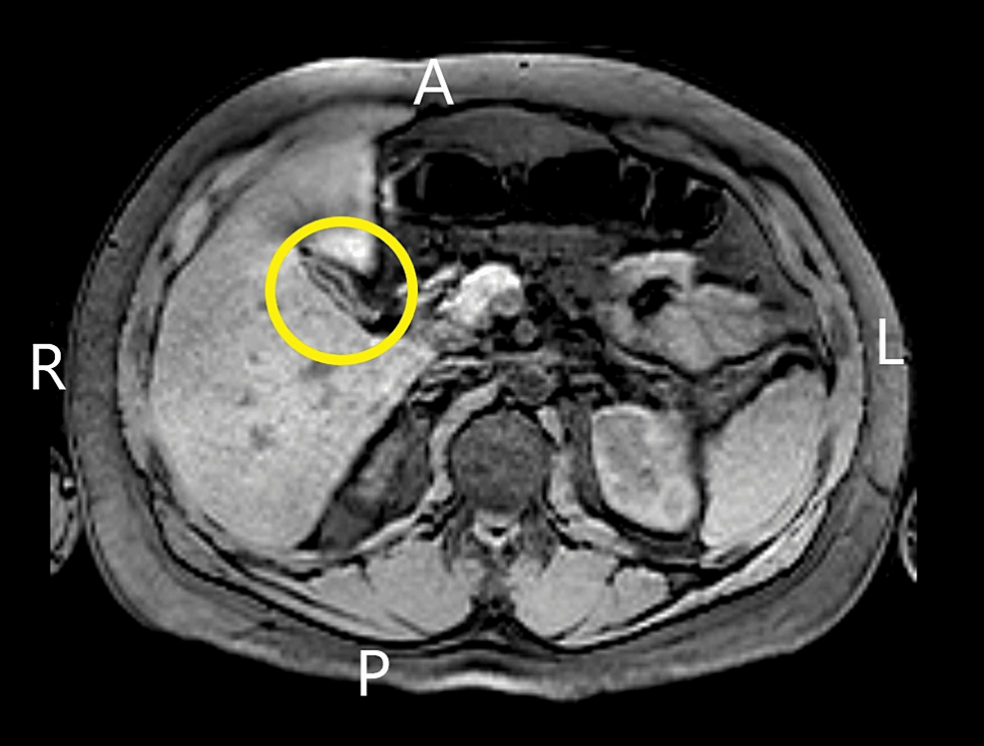
Magnetic resonance cholangiopancreatography (MRCP) A: Anterior; R: Right; L: Left; P: Posterior Yellow Circle: Gallbladder

Point-of-care ultrasound in the emergency department demonstrated gallbladder wall thickening without notable gallstones or common bile duct dilatation and he was empirically initiated on ceftriaxone and metronidazole. Antibiotics were initiated as the patient met sepsis criteria with concern of enteric pathology. Formal abdominal ultrasound later demonstrated normal gallbladder diameter (0.37 cm) (Figure 1) and common biliary duct diameter (0.36 cm) (Figure 2). Further evaluation of his hepatobiliary system with multisequence multiplanar magnetic resonance imaging of the abdomen and pelvis with and without intravenous gadoterate meglumine contrast revealed a collapsed gallbladder with minimal pericholecystic edema, which can be appreciated in the yellow circle in Figure 3. Given his laboratory findings, further workup was performed to evaluate his systemic inflammatory response syndrome including infectious, rheumatic, and metabolic laboratory studies.

Serological markers for hepatitis A, B, and C were negative. Autoimmune studies revealed normal levels of anti-mitochondrial antibody, anti-smooth muscle antibody, and positive double-stranded DNA antibody (titer 1:40). Metabolic markers for inflammatory processes were studied for further investigation revealing highly elevated ferritin (8807 ng/mL). Infectious studies revealed exclusively elevated EBV IgM (158 U/mL) with normal EBV IgG (18.1 U/mL).

After a new, more detailed historical collection, it was observed that the patient had a significant family history of cirrhosis and early mortality. Subsequent genetic testing for hemochromatosis and Wilson's disease was performed and these were negative.

In multidisciplinary discussions with infectious disease teams, antibiotics were stopped and supportive treatment was initiated. He was discharged with prompt follow-up with gastrointestinal teams, which revealed improved acute phase reactants and down-trending aspartate transaminase, alanine transaminase, alkaline phosphatase, and total bilirubin (Table 2).

**Table 2:** Liver Function Tests, Inflammatory Markers, and Immunoglobulin Testing

	Day 1	Day 3	Day 4	Day 5	Day 6	Day 7	Day 18	Reference Range
Aspartate Transaminase (U/L)	247	235	297	274	219	190	93	10-35
Alanine Transaminase (U/L)	282	267	274	271	230	204	138	9-46
Alkaline Phosphatase (U/L)	197	172	175	219	282	322	170	35-144
Total Bilirubin (mg/dL)	3.2	4.6	5.5	6.4	7.4	6.9	1.3	0.2-1.2
Direct Bilirubin (mg/dL)	2.7	4	4.6	5.6	6	5.7	0.7	0.0-0.3
Ferritin (ng/mL)			8807	8165			1405	22-322
EBV IgM (U/mL)			158				97.6	0.0-43.9
EBV IgG (U/mL)			18.1				48.5	0.0-21.9

## Discussion

The acute presentation of this patient's intermittent fevers and right upper quadrant pain guided the initial workup toward acute cholestatic diseases, such as obstructive cholangitis. Upon MRI evaluation, the gallbladder was unremarkable and further history was warranted. Upon re-evaluation and further historical gathering, it was obtained that his father had a history of liver disease and high iron levels. This, in addition to his mixed cholestatic and hepatocellular injury pattern, prompted further investigation into hemochromatosis.

Hemochromatosis is an autosomal recessive disorder, with a prevalence of 1/500 individuals, that predominantly affects white males in their fourth-to-fifth decade [3]. The initial investigation was made with the evaluation of serum ferritin concentrations, which proved to be elevated to 8800. Although the ferritin was elevated, he lacked the early and late manifestations of hemochromatosis, except liver involvement, which prompted further evaluation of his elevated ferritin levels.

Hyperferritinemia is a relatively common finding that prompts further evaluation by a gastroenterologist. Ferritin is a ubiquitous protein that is present in most tissues and functions to bind and oxidize iron, which serves to sequester its use safely within the cell and allows the release of iron according to cellular needs [4]. During severe pathological inflammatory conditions, high levels of serum ferritin are appreciated due to its induction by the acute phase reaction, which reduces free iron available to pathogens [5].

Given his hyperferritinemia, further investigation into viral, bacterial, autoimmune, and malignant etiologies was performed [6]. It was found through this effort that the patient's EBV markers were elevated indicating acute EBV infection. Significantly elevated ferritin in EBV is exceptionally rare and is usually reported with complicated infectious mononucleosis [7], cold-type autoimmune hemolytic anemia [8], and hemophagocytic lymphohistiocytosis [9]. To our knowledge, there has been only one other documented case of hyperferritinemia in the setting of acute EBV-induced hepatitis [10].

EBV commonly presents as a self-limiting infection, mainly as infectious mononucleosis, which presents as fevers, malaise, tonsillitis, and lymphadenopathy; the feared complication from EBV is malignancy. EBV can be evaluated by AST/ALT elevation providing a hepatic picture, but is not usually known to cause hepatitis or painless jaundice [11]. Given the rarity of symptomatic hepatitis and painless jaundice, this diagnosis should be considered in all patients with unexplained hepatitis [12,13].

In our case, we illustrate by laboratory testing, that EBV presented atypically, but resolved in a self-limiting fashion. With the use of serial liver-function testing (Table 1 and Table 2), we not only appreciate the acute-to-chronic immunological transition, but also the transient peaking (Day 4) and decline of AST, ALT, and ferritin markers (Day 18).

Although the treatment of EBV remains supportive, as the condition is self-limiting, the use of steroids has gained increased controversy [14]. Comparatively, antiviral therapy has been used to reduce EBV shedding, but is clinically ineffective [15].

## Conclusions

In this report, our goal was to raise awareness among healthcare professionals about Epstein-Barr acute hepatitis - a rare differential diagnosis for hyperferritinemia without iron overload. The aim is to avoid unnecessary and potentially harmful medical interventions in patients presenting with intermittent fevers and abdominal pain, along with elevated liver function tests. We emphasize the importance of considering infectious causes when dealing with hepatitis, going beyond the conventional evaluation for hepatitis A, B, and C.

Incorporating acute phase reactants can strengthen the suspicion of an inflammatory and/or infectious phase. However, in the presence of hyperferritinemia, it's crucial to rule out hemochromatosis. This underscores the significance of a thorough familial history investigation, especially in the context of early liver disease and death, particularly when confronted with highly elevated and abnormal laboratory testing.

It is worth noting that Epstein-Barr virus (EBV), typically a self-limiting infection requiring supportive care, may, in some instances, manifest severely, necessitating anti-viral and anti-inflammatory interventions. In such cases, clinical judgment plays a pivotal role in guiding appropriate therapeutic decisions.

## References

[1] Hoover K, Higginbotham K (2023). Epstein-Barr Virus. In: StatPearls [Internet].

[2] Crum NF (2006). Epstein Barr virus hepatitis: case series and review. South Med J.

[3] Porter J, Rawla P (2023). Hemochromatosis. In: StatPearls [Internet].

[4] Piperno A, Pelucchi S, Mariani R (2023). Hereditary hyperferritinemia. Int J Mol Sci.

[5] Mantovani A, Garlanda C (2023). Humoral innate immunity and acute-phase proteins. N Engl J Med.

[6] Kushner I (1982). The phenomenon of the acute phase response. Ann N Y Acad Sci.

[7] Thoufeeq MH, Ali Khan SL, Jain SK, Al-Shakerchi H, Hussain M (2007). A case of acute infectious mononucleosis presenting with very high ferritin. World J Gastroenterol.

[8] Dematapitiya C, Perera C, Chinthaka W (2019). Cold type autoimmune hemolytic anemia- a rare manifestation of infectious mononucleosis; serum ferritin as an important biomarker. BMC Infect Dis.

[9] Goudarzipour K, Kajiyazdi M, Mahdaviyani A (2013). Epstein-Barr virus-induced hemophagocytic lymphohistiocytosis. Int J Hematol Oncol Stem Cell Res.

[10] Theodory B, Dopp M, Swisher AR, Flores RM, Robb PM (2023). Epstein-Barr virus induced acute hepatitis with hyperferritinemia: a rare presentation. IDCases.

[11] Bhattacharjee S, Ali A, Sayed S, Siyal T, Das T (2022). An acute presentation of Epstein-Barr virus (EBV) infection in an immunocompromised gentleman. Cureus.

[12] Vine LJ, Shepherd K, Hunter JG (2012). Characteristics of Epstein-Barr virus hepatitis among patients with jaundice or acute hepatitis. Aliment Pharmacol Ther.

[13] Moniri A, Tabarsi P, Marjani M, Doosti Z (2017). Acute Epstein - Barr virus hepatitis without mononucleosis syndrome: a case report. Gastroenterol Hepatol Bed Bench.

[14] Manappallil RG, Mampilly N, Josphine B (2019). Acute hepatitis due to infectious mononucleosis. BMJ Case Rep.

[15] Moniri A, Tabarsi P, Marjani M, Doosti Z (2017). Acute Epstein - Barr virus hepatitis without mononucleosis syndrome: a case report. Gastroenterol Hepatol Bed Bench.

